# Transcription factors Foxa1 and Foxa2 are required for adult dopamine neurons maintenance

**DOI:** 10.3389/fncel.2014.00275

**Published:** 2014-09-09

**Authors:** Andrii Domanskyi, Heike Alter, Miriam A. Vogt, Peter Gass, Ilya A. Vinnikov

**Affiliations:** ^1^Division of Molecular Biology of the Cell I, German Cancer Research Center (DKFZ)Heidelberg, Germany; ^2^RG Animal Models in Psychiatry, Medical Faculty Mannheim, Central Institute of Mental Health, Heidelberg UniversityMannheim, Germany

**Keywords:** Foxa1, Foxa2, dopamine, dopaminergic neurons, transgenic mice, neurodegeneration, substantia nigra, Parkinson's disease

## Abstract

The proteins Foxa1 and Foxa2 belong to the forkhead family of transcription factors and are involved in the development of several tissues, including liver, pancreas, lung, prostate, and the neural system. Both Foxa1 and Foxa2 are also crucial for the specification and differentiation of dopamine (DA) neurons during embryonic development, while about 30% of mice with an embryonic deletion of a single allele of the *Foxa2* gene exhibit an age-related asymmetric loss of DA neurons and develop locomotor symptoms resembling Parkinson's disease (PD). Notably, both Foxa1 and Foxa2 factors continue to be expressed in the adult dopamine system. To directly assess their functions selectively in adult DA neurons, we induced genetic deletions of Foxa1/2 transcription factors in mice using a tamoxifen inducible tissue-specific CreERT2 recombinase expressed under control of the dopamine transporter (DAT) promoter (DATCreERT2). The conditional DA neurons-specific ablation of both genes, but not of *Foxa2* alone, in early adulthood, caused a decline of striatal dopamine and its metabolites, along with locomotor deficits. At early pre-symptomatic stages, we observed a decline in aldehyde dehydrogenase family 1, subfamily A1 (Aldh1a1) protein expression in DA neurons. Further analyses revealed a decline of aromatic amino acid decarboxylase (AADC) and a complete loss of DAT expression in these neurons. These molecular changes ultimately led to a reduction of DA neuron numbers in the substantia nigra pars compacta (SNpc) of aged *cFoxa1/2*^−/−^ mice, resembling the progressive course of PD in humans. Altogether, in this study, we address the molecular, cellular, and functional role of both Foxa1 and Foxa2 factors in the maintenance of the adult dopamine system which may help to find better approaches for PD treatment.

## Introduction

Parkinson's disease (PD) is one of the most prevalent age-related movement disorders occurring in about 1% of the population above the age of 60 (Abou-Sleiman et al., 2006; Ferri et al., 2007). PD affects multiple neuronal systems (Braak et al., 2003), however, the major motor symptoms are caused by the degeneration of dopamine (DA) neurons in the substantia nigra pars compacta (SNpc) (Moore et al., 2005). Current treatment strategies are providing only symptomatic relief to the patients and are not able to prevent the progression of neurodegeneration. Therefore, the identification and characterization of the mechanisms involved in the maintenance of adult DA neurons is critically important for the advance of novel therapies for PD (Meissner et al., 2011).

The development of midbrain DA neurons is a highly orchestrated process involving coordinated action of multiple signaling molecules and transcription factors, such as Shh, Wnt, Otx2, Pitx3, Nurr1 (Nr4a2), Foxa1, and Foxa2 (Perlmann and Wallen-Mackenzie, 2004; Ferri et al., 2007; Smidt and Burbach, 2007; Omodei et al., 2008; Jacobs et al., 2009; Joksimovic et al., 2009; Mesman et al., 2014). Several of these factors continue to be expressed in post-mitotic and also in adult DA neurons contributing to the functional maintenance of this neuronal population. For example, Otx2 expressed in the ventral tegmental area (VTA) DA neurons controls the identity of this neuronal subtype and confers its resistance to 1-methyl-4-phenyl-1,2,3,6-tetrahydropyridine (MPTP) (Di Salvio et al., 2010; Di Giovannantonio et al., 2013). A selective ablation of orphan nuclear receptor Nurr1 in adult DA neurons using a tamoxifen (TAM)-inducible Cre/LoxP recombination system leads to the fiber pathology of this neuronal population and loss of striatal dopamine, recapitulating early stages of PD development (Kadkhodaei et al., 2009). It has recently been shown that Nurr1 regulates the expression of nuclear-encoded mitochondrial genes and is involved in sustaining high respiratory function in adult DA neurons (Kadkhodaei et al., 2013). Transcription factors Foxa1 and Foxa2 play crucial roles not only during the early development and specification (Ferri et al., 2007; Kittappa et al., 2007; Moore et al., 2005), but also in the process of maturation of DA neurons, as has been demonstrated by deleting these factors in post-mitotic DA neurons during late embryonic development (Stott et al., 2013). Interestingly, an asymmetrical degeneration of SNpc DA neurons has been observed in about 30% of aged mice bearing a global heterozygous deletion of *Foxa2* gene allele (Kittappa et al., 2007).

Proteins belonging to Foxa family (Foxa1, Foxa2, and Foxa3) share very high sequence homology within the DNA binding domain, whereas outside of this region they are less similar, and Foxa3 being shorter and more divergent from Foxa1/2 (Lai et al., 1991; Friedman and Kaestner, 2006; Kaestner, 2010). The loss-of-function studies demonstrate that Foxa1 and Foxa2 have partially overlapping functions during embryonic development of DA neurons; both Foxa1 and Foxa2 factors are required for the expression of Lmx1a, Lmx1b (Lin et al., 2009), Nurr1 and engrailed 1 (En1) (Ferri et al., 2007) in immature DA neurons and for the expression of AADC and TH in early post-mitotic DA neurons (Ferri et al., 2007; Stott et al., 2013). Consequently, a combined deletion of Foxa1 and Foxa2 in embryonic DA neurons results in reduced binding of Nurr1 to *Th* and *Aadc* gene promoters leading to a significant loss of TH and AADC expression in the SNpc of embryos and adult mice (Stott et al., 2013).

The expression of both Foxa1 and Foxa2 continues into adulthood (Kittappa et al., 2007; Stott et al., 2013), suggesting that, in addition to their essential role in the development, specification and maturation, both proteins are also involved in the physiological functions of adult DA neurons.

The deregulation of Foxa1/2 may also contribute to demise of DA neurons during PD progression in humans. Indeed, by searching the online databases, such as the National Center for Adult Stem Cell Research Parkinson's review database (Sutherland et al., 2009) and ParkDB (Taccioli et al., 2011) that contain manually curated, re-analyzed and annotated microarray datasets from PD patients and PD models, we found several datasets showing the down-regulation of Foxa1 and Foxa2 expression in the SNpc of PD patients (Hauser et al., 2005; Zhang et al., 2005; Moran et al., 2006; Lesnick et al., 2007).

However, no previous studies have directly addressed the role of Foxa1/2 factors in adult DA neurons. Here we used a tissue-specific TAM-inducible Cre recombination to ablate both the *Foxa1* and *Foxa2* genes selectively in adult DA neurons. This deletion resulted in DA neurons losing their dopaminergic phenotype, which was reflected by the decline in expression of Aldh1a1, AADC, DAT and TH, as well as reduced striatal dopamine leading to the development of locomotor abnormalities, and, ultimately, loss of the neurons in aged *cFoxa1/2*^−/−^ double knockout mice.

## Materials and methods

### Animal experiments

Mice were maintained in the C57Bl/6N genetic background on a 12 h light-dark cycle with free access to water and food. The *Foxa2^fl/fl^DATCreERT2, Foxa1^fl/wt^Foxa2^fl/fl^DATCreERT2, Foxa1^fl/fl^Foxa2^fl/wt^DATCreERT2*, and *Foxa1^fl/fl^Foxa2^fl/fl^DATCreERT2* mouse lines (referred hereafter as *cFoxa2*^−/−^, *cFoxa1*^+/−^*/2*^−/−^, *cFoxa1*^−/−^*/2*^+/−^, and *cFoxa1/2*^−/−^, respectively) were generated by mating *Foxa1^fl/fl^* (Gao et al., 2008) and *Foxa2^fl/fl^* mice (Sund et al., 2000) with *DATCreERT2* (Engblom et al., 2008) mice. Inducible Cre recombinase was activated in 8–10 week-old mice by intraperitonial injections of 1 mg tamoxifen (TAM, Sigma-Aldrich) diluted in sunflower oil twice daily for five consecutive days (Domanskyi et al., 2011; Rieker et al., 2011; Vinnikov et al., 2014). Littermates harboring only floxed alleles were used as controls. All experimental procedures were performed with the approval by the institutional Committee on Ethics of Animal Experimentation and carried out in accordance with the local and European legislation on the protection of animals used for scientific purposes.

### Histological analyses

Mice at the indicated time points after TAM injections (post-TAM) were perfused with 4% paraformaldehyde (PFA); the brains were dissected and fixed overnight in 4% PFA and processed for either paraffin or vibratome sections. Upon dissection, no differences in morphology, weight, or size of the brains were observed in *cFoxa1/2* animals compared to control littermates. Immunohistochemical and immunofluorescent stainings were performed as previously described (Domanskyi et al., 2011; Rieker et al., 2011) using the following antibodies: anti-tyrosine hydroxylase (TH) (1:1000, Millipore #AB1542), anti-aromatic amino acid decarboxylase (AADC) (1:1000, Millipore #AB1569), anti-DAT (1:500, Millipore #MAB369), anti-aldehyde dehydrogenase 1 family, member A1 (Aldh1a1) (1:100, Abcam #ab52492). Fluorescent signals in the brain samples were visualized directly with confocal system TSC SP5 (Leica) or LSM780 (Zeiss).

Quantification of the Aldh1a1- and TH-positive cells on immunostained brain sections was performed either by blinded experimenters or by using MCID Image Analysis software (InterFocus Imaging) as previously described (Isermann et al., 2007; Domanskyi et al., 2011). The SNpc and the VTA were identified according to the anatomical landmarks (Zaborszky and Vadasz, 2001) and the neurons were counted for each mouse from at least five sections covering the ventral midbrain (Domanskyi et al., 2011). For immunofluorescently stained samples, number of TH-positive neurons in the SNpc and VTA in single confocal plane images was determined by blinded investigators, followed by a quantification of the percentage of Aldh1a1-positive neurons within the TH-positive population on the same sections. Quantification of immunohistochemically stained TH-positive neurons in aged *cFoxa1/2*^−/−^mice was performed in the same way, except that the region of interest was limited by the SNpc and that counting was performed automatically by MCID Image Analysis software.

### Quantitative RT-PCR

Total RNA isolated from ventral midbrain samples served as a template for DNA synthesis using Super-Script III first-strand synthesis kit (Invitrogen). For genomic DNA contamination control, samples with no added reverse transcriptase enzyme were included. Quantitative PCR was performed with a CFX96 Real-Time System (Bio-Rad) using TagMan Gene Expression Assays (Life Technologies) according to the manufacturer's instructions. The mRNA levels of *Hprt1* were measured to control for the equal amount of input cDNA. The following probes were used for detection of *En1, Foxa1, Foxa2, Hprt1, Lmx1b, Nr4a2, Pitx3, Th*, and *Ucp2*: Mm00438709_m1, Mm00484713_m1, Mm00839704_mH, Mm01545399_m1, Mm00440209_m1, Mm00443056_m1, Mm01194166_g1, Mm00447557_m1, and Mm00495907_g1, respectively.

### Behavioral assays

The accelerating and constant speed rotarod assays were performed as previously described (Domanskyi et al., 2011; Rieker et al., 2011). Briefly, for the constant speed rotarod assay mice were initially trained to attain stable baseline levels of performance staying on the rod rotating at 15 rpm for 60 s. After that, the mice received several trials at 25 and 35 rpm rotation speed with 60 s maximum trial length and 5 min intervals between individual trials. Two maximal values per speed per day were used to calculate the average which was used for subsequent statistical analyses. This setting successfully corrects for effects unrelated to motor/balance performance such as re-learning, fatigue, tendencies to learned helplessness or over-performance/hyperactivity.

For the open field test, mice were placed individually into the open arena and monitored for 5 min by a video camera. The resulting data were analyzed using the image processing systems EthoVision 3.0 (Noldus Information Technology) (Chourbaji et al., 2008) and Any-maze 4.82 (Stoelting Co.). For each sample, the systems recorded position, object area and the status of defined events.

### Measurements of striatal dopamine and its metabolites

After decapitation, the striata were rapidly dissected on ice, weighed, and frozen on dry ice. Measurements of striatal dopamine, 3,4-dihydroxyphenylacetic acid (DOPAC) and homovanillic acid (HVA) were performed by reverse-phase HPLC with electrochemical detection method (HPLC-ED) as previously described (Otto and Unsicker, 1990; Enkel et al., 2014).

### Statistical analyses

Statistical significance was calculated by Student's two-tailed unpaired *t*-test or Two-Way ANOVA followed by Bonferroni *post-hoc* test using GraphPad Prism software (GraphPad Scientific, USA). *p* values less than 0.05 were considered significant (^*^p < 0.05; ^**^p < 0.01; ^***^p < 0.001) with respect to control groups. Data in text and figures are represented as means ± s.e.m.

## Results

### Foxa2 deletion in adult dopamine neurons does not lead to neurodegeneration

**Figure 1:**
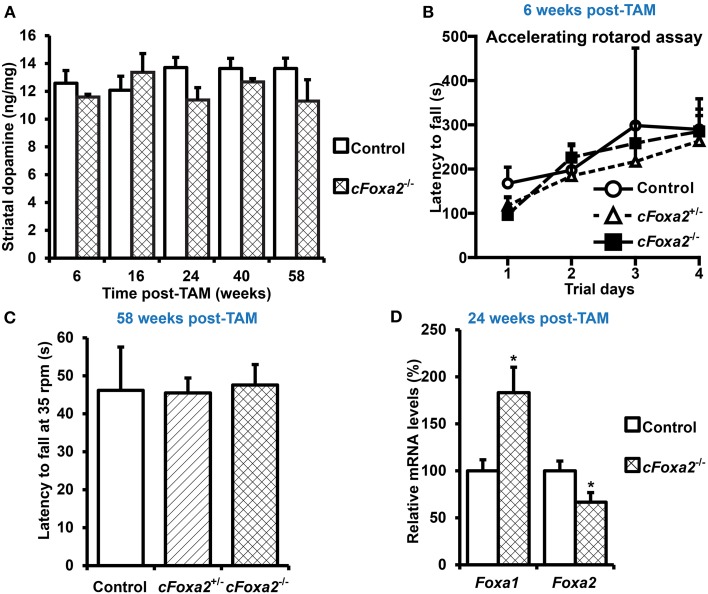
**Absence of changes in locomotor activity and striatal dopamine content upon conditional ablation of *Foxa2* gene in adult dopamine neurons. (A)** Striatal dopamine content in *cFoxa2*^−/−^ mice at indicated time points after TAM treatment (post-TAM). *n* = 4, 3, 3, 7, 14, 10, 6, 6, 5, 3 for the groups from left to the right on the graph. **(B)** Latency to fall in the accelerating rotarod assay in control, *cFoxa2*^+/−^, and *cFoxa2*^−/−^ mice 6 weeks post-TAM (*n* = 4, 3, 3, respectively). **(C)** Latency to fall in the constant speed (35 rpm) rotarod assay in aged control, *cFoxa2*^+/−^, and *cFoxa2*^−/−^ mice 58 weeks post-TAM (*n* = 3, 15, 9, respectively). **(D)** Reciprocal change of *Foxa1* and *Foxa2* mRNA levels in the ventral midbrain of control (*n* = 10) and *cFoxa2*^−/−^ (*n* = 5) mice 24 weeks post-TAM. ^*^p < 0.05 in comparison to control, as determined by Student's unpaired *t*-test.

To directly investigate the role of Foxa2 in maintenance of adult DA neurons, we crossed *Foxa2^fl/fl^* mice (Sund et al., 2000) with the *DATCreERT2* line. These transgenic mice provide a tight spatial and temporal control of recombination upon treatment with estrogen receptor antagonist TAM (Engblom et al., 2008). In *cFoxa2*^−/−^ mice harboring deletions of both *Foxa2* alleles in adult DA neurons, no sign of neurodegeneration phenotype was observed at any time point tested (Figures 1A–C). Both striatal dopamine content (Figure 1A) and motor functions, measured in the accelerated rotarod assay (Figure 1B) or in the more sensitive (Monville et al., 2006; Brooks and Dunnett, 2009) constant speed rotarod assay (Figure 1C), were at normal levels in *cFoxa2*^−/−^ mice up to 58 weeks after recombination onset by TAM treatment (post-TAM) (Figures 1A,C).

The previous works delineating the role of Foxa factors in the liver, pancreas and developing DA neurons (Ferri et al., 2007; Kaestner, 2010; Stott et al., 2013) have detected striking similarities in binding motifs, regulated genes and global functions of Foxa1 and Foxa2 factors, indicating that Foxa1 could compensate for the function of Foxa2 in adult DA neurons of *cFoxa2*^−/−^ mice. Indeed, despite significant down-regulation of *Foxa2* mRNA levels in the ventral midbrain of *cFoxa2*^−/−^ mice, the levels of *Foxa1* in these animals were up-regulated (Figure 1D). These results imply the existence of a functional overlap between Foxa1 and Foxa2 in adult DA neurons.

### Foxa1/2 deletion in adult dopamine neurons causes locomotor deficits

**Figure 2:**
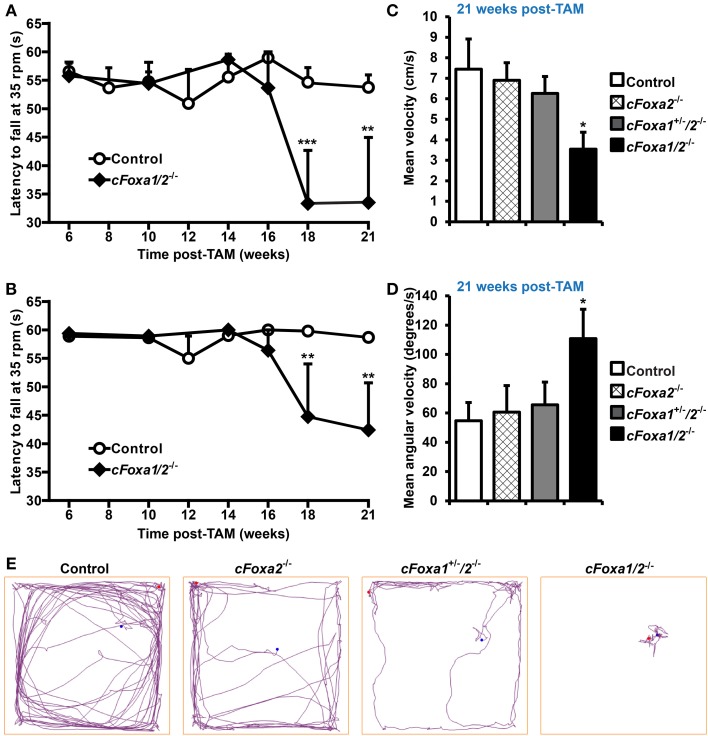
**Locomotor impairments in *cFoxa1/2*^−/−^ double mutant mice**. (**A,B**) Latency to fall in the constant speed rotarod assay at 35 rpm **(A)** and 25 rpm **(B)** in control and *cFoxa1/2*^−/−^ mice (*n* = 13 and 5, respectively) at indicated time points post-TAM **(C–E)** The quantification of mean velocity **(C)** and mean angular velocity **(D)**, and representative running tracks **(E)** of control, *cFoxa2*^−/−^, *cFoxa1*^+/−^*/2*^−/−^, and *cFoxa1/2*^−/−^ mice (*n* = 11, 6, 10, 5, respectively) in the open field assay performed 21 weeks post-TAM. Length of the open field box side, 60 cm. ^*^p < 0.05, ^**^p < 0.01, ^***^p < 0.001 in comparison to control, as determined by Student's unpaired *t*-test or Two-Way ANOVA followed by Bonferroni *post-hoc* test. Blue and red dots represent, respectively, the initial and final position of a mouse in the open field assay.

In order to conditionally ablate both Foxa1 and Foxa2 factors in adult DA neurons, we created *cFoxa1/2*^−/−^ mice by crossing *Foxa1^fl/fl^* (Gao et al., 2008) and *Foxa2^fl/fl^* (Sund et al., 2000) with *DATCreERT2* (Engblom et al., 2008) animals. The quantitative PCR (qPCR) analysis showed that both *Foxa1* and *Foxa2* mRNA expression in the ventral midbrain decreased in *cFoxa1/2*^−/−^ mice (Table 1), confirming the successful deletion of Foxa1 and Foxa2 in these animals. Starting from the 18th week post-TAM, *cFoxa1/2*^−/−^ mice developed locomotor impairments, as determined by the constant speed rotarod assay (Figures 2A,B). Importantly, when tested at 21 weeks post-TAM, the mice exhibited a decreased activity in the open field test paralleled by an increased circling behavior (Figures 2C–E). The asymmetric circling behavior (Figure 2D) in *cFoxa1/2*^−/−^ mice may partly be due to an increased difference in dopamine levels in left and right brain hemispheres (Supplementary Figure 1). Moreover, at 24 weeks post-TAM we observed a significant reduction of the striatal content of dopamine and its metabolites, DOPAC and HVA (Table 2) in *cFoxa1/2*^−/−^ mice.

**Table 1:** **Relative levels of *Foxa1* and *Foxa2* mRNA in the ventral midbrain of mice 24 weeks after conditional ablation of these factors in adult dopamine neurons**.

**Line name**	**Genotype**	***n***	***Foxa1* (%)**	***Foxa2* (%)**
Control	*Foxa1^fl/fl^Foxa2^fl/fl^*	10	100 ± 11.64	100 ± 10.36
*cFoxa2*^−/−^	*Foxa2^fl/fl^DATCreERT2*	5	183.24 ± 27.05^*^	66.56 ± 10.21^*^
*cFoxa1*^+/−^*/2*^−/−^	*Foxa1^fl/wt^Foxa2^fl/fl^DATCreERT2*	10	122.32 ± 19.94	61.1 ± 8.24^**^
*cFoxa1/2*^−/−^	*Foxa1^fl/fl^Foxa2^fl/fl^DATCreERT2*	5	54.93 ± 13.04^*^	68.25 ± 7.35^*^

The data, presented as mean values ± s.e.m., are normalized to the Hprt1 levels and expressed as percentage relative to control;

* **p* < 0.05*,

** **p* < 0.01 in comparison to control, as determined by Student's unpaired t-test*.

**Table 2:** **Levels of dopamine and its metabolites in the striata of mice 24 weeks after conditional ablation of Foxa1/2 factors in adult dopamine neurons**.

**Line name**	***n***	**Dopamine (ng/mg)**	**DOPAC (ng/mg)**	**HVA (ng/mg)**
Control	10	13.49 ± 1.0	0.87 ± 0.06	1.24 ± 0.12
*c*Foxa2^−/−^	6	12.8 ± 0.9	0.86 ± 0.06	1.16 ± 0.09
*c*Foxa1^+/−^/2^−/−^	10	11.92 ± 0.78	0.67 ± 0.04^*^	1.02 ± 0.07
*c*Foxa1/2^−/−^	5	6.27 ± 0.76^***^	0.38 ± 0.05^***^	0.67 ± 0.11^**^

The data, presented as mean values ± s.e.m., are normalized to the striatal weight;

* **p* < 0.05*,

** **p* < 0.01*,

*** **p* < 0.001 in comparison to control, as determined by Student's unpaired t-test. DOPAC, 3,4-dihydroxyphenylacetic acid; HVA, homovanillic acid*.

### Loss of Aldh1a1 expression in the ventral midbrain precedes the onset of locomotor impairments in *cFoxa1/2^−/−^* mice

**Figure 3:**
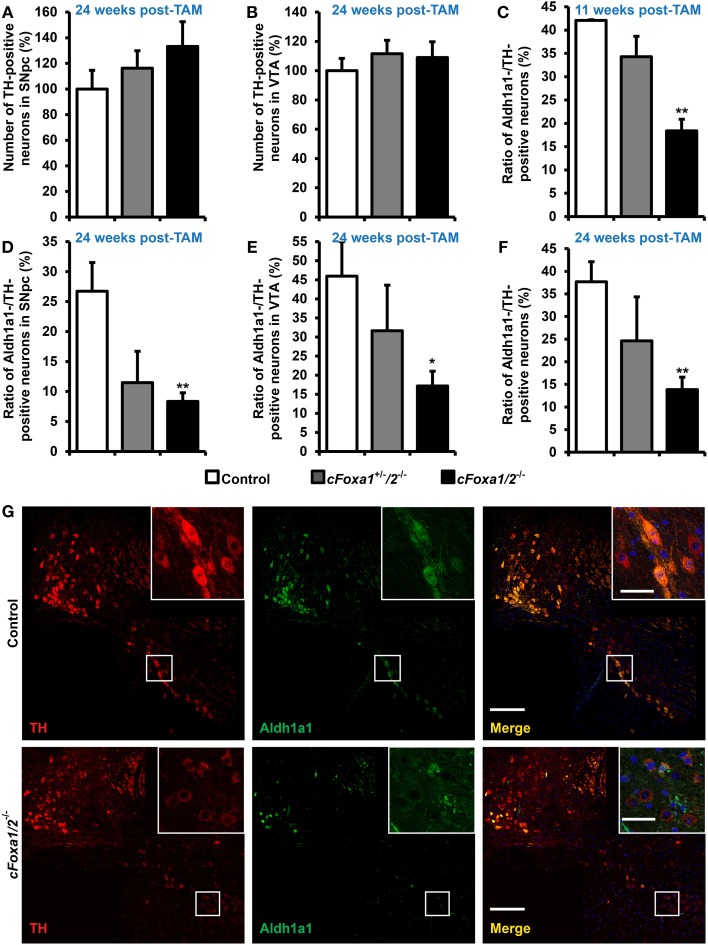
**Loss of the Aldh1a1 expression in adult dopamine neurons precedes the onset of locomotor impairments in *cFoxa1/2*^−/−^ mice. (A,B)** Quantification of tyrosine hydroxylase (TH)-positive neurons in the SNpc **(A)** or in the VTA **(B)** of control, *cFoxa1*^+/−^*/2*^−/−^, and *cFoxa1/2*^−/−^ mice 24 weeks post-TAM (*n* = 4, 3, and 5, respectively). **(C–F)** Quantification of aldehyde dehydrogenase 1 family, member A1 (Aldh1a1)-positive neurons in the ventral midbrain **(C,F)**, SNpc **(D)** or VTA **(E)** of control, *cFoxa1*^+/−^*/2*^−/−^, and *cFoxa1/2*^−/−^ mice 11 weeks **(C)** and 24 weeks **(D–F)** post-TAM expressed relative to the number of TH-positive neurons (*n* = 4, 3, and 5, respectively). **(G)** Representative microphotographs of TH (red) and Aldh1a1 (green) immunofluorescent staining and co-localization of these proteins and DAPI (blue) in the ventral midbrain sections from control and *cFoxa1/2*^−/−^ mice 24 weeks post-TAM. Scale bar, 200 μm for overviews and 50 μm for insets. ^*^p < 0.05, ^**^p < 0.01 in comparison to control, as determined by Student's unpaired *t*-test.

**Figure 4:**
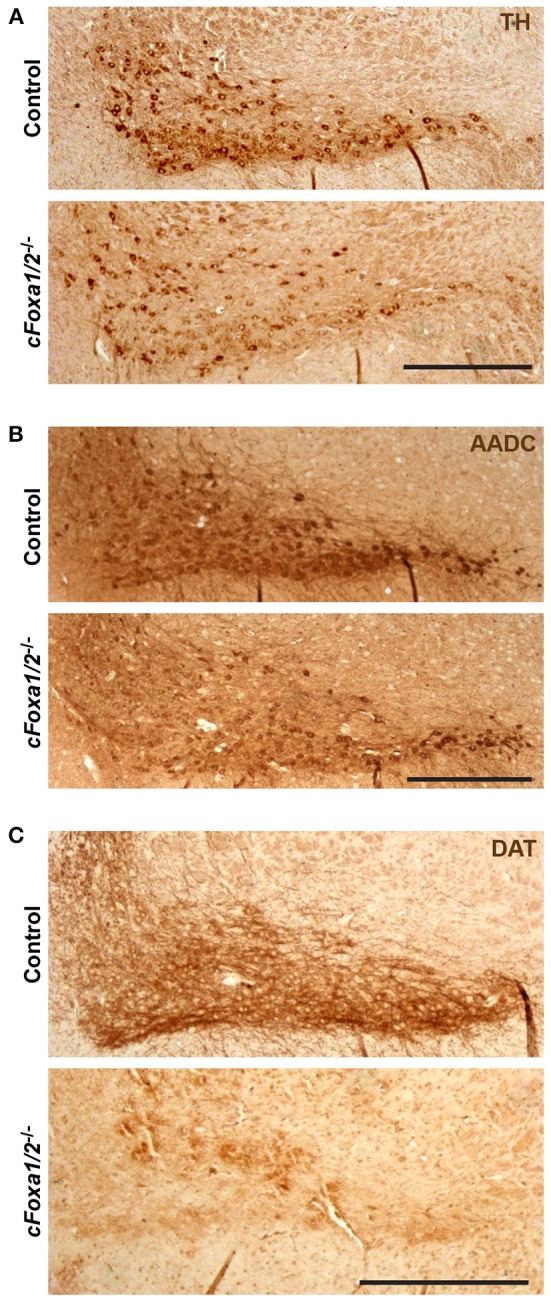
**Down-regulation of dopamine neuronal markers in *cFoxa1/2*^−/−^ mice. (A–C)** Representative microphotographs of tyrosine hydroxylase (TH) **(A)**, aromatic amino acid decarboxylase (AADC) **(B)** and dopamine transporter (DAT) **(C)** immunostaining in the ventral midbrain of control and *cFoxa1/2*^−/−^ mice 24 weeks post-TAM. Scale bar, 500 μm.

At the same time point, 24 weeks post-TAM, the numbers of TH-positive neurons both in the SNpc and in the VTA of *cFoxa1/2*^−/−^ mice were normal (Figures 3A,B). However, already at 11 weeks post-TAM, we observed a dramatic decrease of the numbers of Aldh1a1-positive DA neurons (Figure 3C) that was even more evident at 24 weeks and was stronger in the SNpc than in the VTA (Figures 3D–G). Aldh1a1 is neuroprotective in DA neurons (Anderson et al., 2011; Wey et al., 2012; Liu et al., 2014) and, therefore, decline in its expression may render adult DA neurons toward degeneration. Accordingly, even though the numbers of TH-positive neurons at 24 weeks post-TAM *cFoxa1/2*^−/−^ mice did not change, these neurons exhibited a decrease in the ventral midbrain expression of the key proteins in the DA metabolism: TH, AADC, and especially DAT, as detected in immunostaining experiments (Figures 4A–C). Furthermore, at 24 weeks post-TAM, when we analyzed the expression of several transcripts important for development and functionality of DA neurons (Smidt and Burbach, 2007), we observed a tendency towards decrease of *En1* and *Th* (Supplementary Figure 2). This data suggests that Foxa1/2 proteins are essential in maintaining the expression of crucial factors in adult DA neurons. Indeed, Foxa2 has previously been shown to regulate En1 expression (Ferri et al., 2007). Moreover, it can directly bind to *Th* gene promoter and cooperate with Nurr1 in regulating the expression of TH and AADC in the ventral midbrain (Lee et al., 2010; Stott et al., 2013). Interestingly, the mRNA level of *Ucp2* encoding a mitochondrial uncoupling protein was up-regulated in *cFoxa1/2*^−/−^ mice (Supplementary Figure 2). Overexpression of Ucp2 has been shown to decrease mitochondrial production of reactive oxygen species (Andrews et al., 2005) and protect DA neurons from MPTP (Conti et al., 2005), and *Ucp2* mRNA up-regulation may indicate the existence of a compensatory mechanism which might be activated to protect mitochondrial function in *cFoxa1/2*^−/−^ mice.

**Figure 5:**
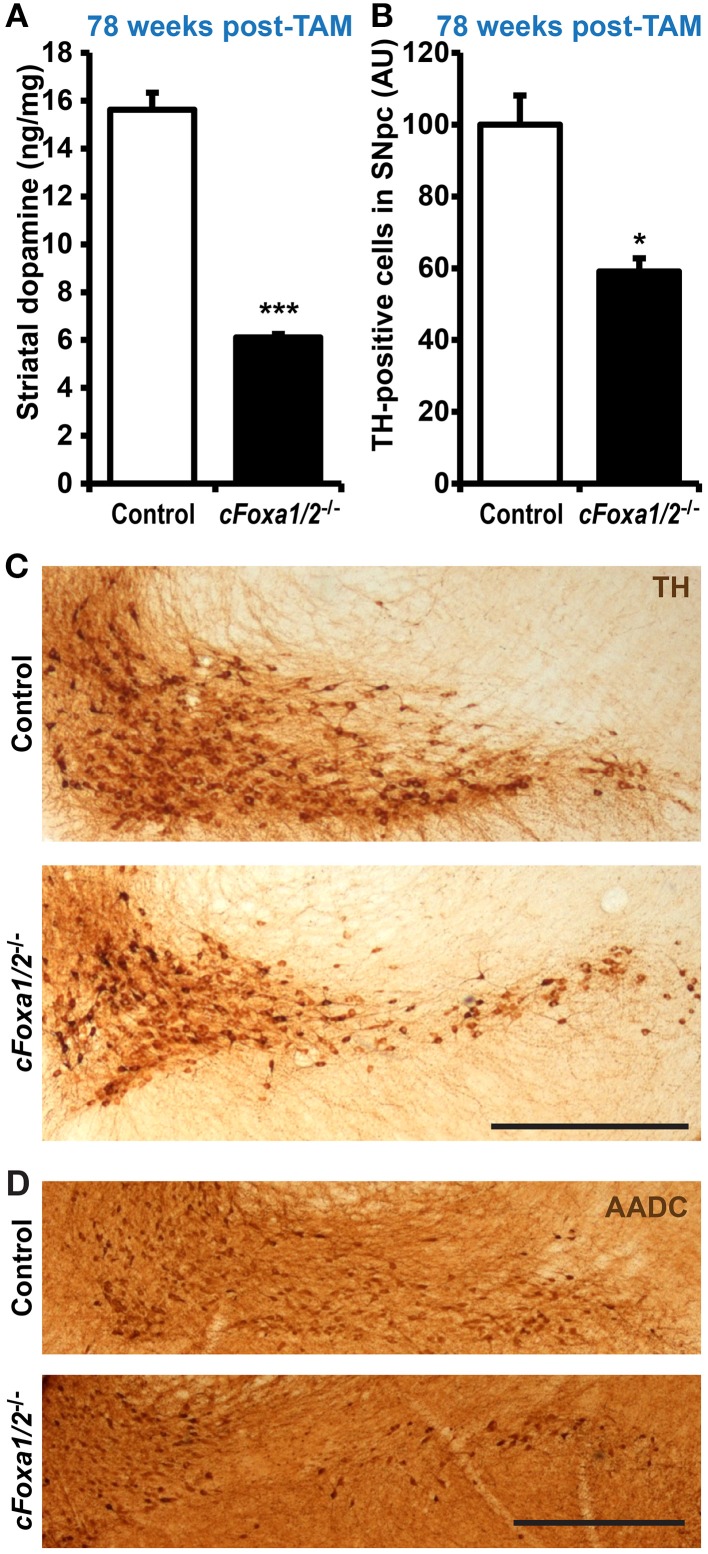
**Loss of TH-positive adult dopamine neurons in aged *cFoxa1/2*^−/−^ mice. (A)** Striatal dopamine content in control or *cFoxa1/2*^−/−^ mice 78 weeks after TAM treatment (*n* = 6 and 3, respectively). **(B)** Quantification of tyrosine hydroxylase (TH)-positive neurons in the SNpc of control and *cFoxa1/2*^−/−^ mice at the same time point (*n* = 3). **(C,D)** Representative microphotographs of TH **(C)** and aromatic amino acid decarboxylase (AADC) **(D)** immunostaining in the ventral midbrain of control and *cFoxa1/2*^−/−^ mice. Scale bar, 500 μm. ^*^p < 0.05, ^***^p < 0.001 in comparison to control, as determined by Student's unpaired *t*-test.

Ultimately, we observed a down-regulation of striatal dopamine (Figure 5A) and the loss of TH-positive neurons in the ventral midbrain of aged *cFoxa1/2*^−/−^ mice 78 weeks post-TAM, visualized by TH and AADC immunostaining and quantification of the TH-positive neurons in the SNpc (Figures 5B–D). Thus, in the early adulthood of *cFoxa1/2*^−/−^ mice, when no TH-positive neurons loss has been yet apparent, a decrease in Aldh1a1 (Figures 3C–G) and, later, in TH and AADC expression (Figures 3G, 4A,B), and a dramatic loss of DAT (Figure 4C) predetermined the fatal outcome for the dopamine system in aged *cFoxa1/2*^−/−^ animals (Figure 5). Considering that Foxa factors can bind to the 5′-regions of *Aldh1a1, Aadc*, and *Th* genes (Lee et al., 2010; Soccio et al., 2011; Stott et al., 2013; Yang et al., 2013), changes in their transcription levels likely represent a molecular mechanism by which Foxa1/2 factors protect adult DA neurons.

The data presented above demonstrate that the deletion of Foxa1/2 ultimately led to a loss of TH-positive neurons in adult mice. To find out whether these results are also clinically relevant, we decided to check if the down-regulation of Foxa1/2 factors and their target genes has been observed in other PD animal models and in PD patients. Online databases of gene expression profiling in clinical PD samples and animal models, namely National Center for Adult Stem Cell Research Parkinson's review database (Sutherland et al., 2009) and ParkDB (Taccioli et al., 2011) provide a comprehensive and constantly updated resource for data mining in a PD-related context. The ParkDB database also allows cross-species comparison of human and mouse expression profiling data. Thus, we searched online both of these databases for the expression changes of *Foxa1, Foxa2, Aldh1a1, Nr4a2, Aadc, Th, En1*, and *Slc6a3*. The levels of these mRNAs were down-regulated in several studies comparing expression profiles of the SNpc samples from PD patients and age-matched healthy subjects (Hauser et al., 2005; Zhang et al., 2005; Moran et al., 2006; Lesnick et al., 2007), indicating that not only Foxa1/2 factors, but also several Foxa1/2-regulated genes are affected in PD patients. Interestingly, while the down-regulation of Foxa1/2 has been observed in the SNpc, the levels of these factors did not significantly change in other tissues of PD patients, such as putamen, cerebellum, occipital cortex (Vogt et al., 2006) and whole blood (Scherzer et al., 2007), possibly indicating a specific role of Foxa factors in the SNpc neurons. Notably, the levels of Foxa1/2 also did not change in the SNpc of transgenic mice overexpressing Hsp70 and alpha-synuclein (dataset E-GEOD-4758) (Klucken et al., 2004), suggesting that Foxa1/2 factors might not be contributing to alpha-synuclein-induced pathology.

## Discussion

Both Foxa1 and Foxa2 are crucial for the specification and development of DA neurons, as was demonstrated by inactivation of these transcription factors prenatally (Ferri et al., 2007; Kittappa et al., 2007; Stott et al., 2013). Moreover, about 30% of mice heterozygous for Foxa2 develop asymmetric loss of DA neurons in the SNpc late in life (Kittappa et al., 2007). First, we sought to study the role of Foxa2 in adult DA neurons by conditionally inactivating the *Foxa2* gene by a DA neuron-specific inducible CreERT2 recombination (Engblom et al., 2008; Domanskyi et al., 2011; Rieker et al., 2011). However, the ablation of only Foxa2 in the presence of intact Foxa1 alleles led neither to neurodegeneration nor to locomotor impairments in *cFoxa2*^−/−^ mice (Figures 1A–C). Moreover, we observed an up-regulation of *Foxa1* mRNA in the ventral midbrain samples from *cFoxa2*^−/−^ mice (Figure 1D). This up-regulation may be caused by a yet undiscovered feedback loop mechanism to compensate for the function of Foxa2 in adult DA neurons, explaining the absence of phenotype in *cFoxa2*^−/−^ mice. Indeed, Foxa1 and Foxa2 have been reported to regulate the development of DA neurons in a dose-dependent manner (Ferri et al., 2007). Both Foxa1 and Foxa2 are important “pioneering” factors which open the chromatin for binding of other transcription regulators (Friedman and Kaestner, 2006). Possibly, the Foxa factors have evolved to compensate for the down-regulation of either protein.

In agreement with the redundant functions of Foxa factors, locomotor impairments and movement asymmetry developed only after conditional inactivation of both Foxa1 and Foxa2, but not Foxa2 alone, in adult DA neurons (Figures 2A–E). Similar phenotype was observed in our previous studies after conditional ablation of polymerase I transcription initiation factor Ia (TifIa) in adult DA neurons (Domanskyi et al., 2011; Rieker et al., 2011). In *cFoxa1/2*^−/−^ mice, deficits in locomotion became apparent 18 weeks after Foxa1/2 ablation when the double mutant mice exhibited a reduced latency to fall off the rotarod. In the open field test performed 21 weeks post-TAM, *cFoxa1/2*^−/−^ mice showed locally restricted but highly increased circling behavior with almost no forward locomotion. Both behaviors reflect bradykinesia and asymmetric movements, symptoms, the onset of which becomes apparent upon loss of striatal DA content in patients with PD (Taylor et al., 2010). Especially a circling behavior is a classical symptom of unbalanced DA levels in PD, and it is often induced by unilateral lesions (Heuer et al., 2012).

In *cFoxa1/2*^−/−^ mice, there was no apparent loss of TH-positive neurons either in the SNpc or in the VTA (Figures 3A–B) up to 24 weeks after Foxa1/2 ablation. However, we detected a significant down-regulation of Aldh1a1 in TH-positive DA neurons of the ventral midbrain of *cFoxa1/2*^−/−^ mice at 11 weeks after recombination (Figure 3C), that became even more pronounced at 24 weeks, with TH-positive DA neurons in the SNpc being more affected than those in the VTA (Figures 3C–G). Aldh1a1 catalyses the oxidation of 3,4-dihydroxyphenylacetaldehyde (DOPAL) to DOPAC which is then converted to HVA in DA neurons (Marchitti et al., 2007). Thus, down-regulation of Aldh1a1 may also contribute to reduced levels of both these dopamine metabolites that occurred in *cFoxa1/2*^−/−^ mice 24 weeks after recombination (Table 2). A protective function of Aldh1a1 in DA neurons has been reported in several studies (Anderson et al., 2011; Wey et al., 2012; Liu et al., 2014); moreover, reduced Aldh1a1 expression and the loss of Aldh1a1-positive DA neurons has been observed in post-mortem brain sections from PD patients (Liu et al., 2014). Interestingly, chromatin immunoprecipitation (ChIP) studies identified two Foxa2 binding sites 5′ to the protein coding sequence of human and mouse *Aldh1a1* gene (Soccio et al., 2011; Yang et al., 2013), suggesting that Foxa factors may directly regulate Aldh1a1 expression. Concomitant with the down-regulation of striatal dopamine, the observed reduction in Aldh1a1 expression was the earliest molecular manifestation of functional disturbances in DA neurons detectable 11 weeks after the conditional ablation of Foxa factors.

Interestingly, even though the number of TH-positive neurons in *cFoxa1/2*^−/−^ mice at 24 weeks after recombination did not change, the TH immunostaining intensity in the ventral midbrain of *cFoxa1/2*^−/−^ mice was lower than that of controls (Figures 3G, 4A) and the *Th* mRNA levels also had a tendency towards a decrease (Supplementary Figure 2). We observed even more pronounced loss of immunostaining intensity for two other markers of DA neurons, AADC and DAT (Figures 4B,C), paralleled by altered morphology of these cells (Figure 3G), suggesting that the ablation of Foxa1 and Foxa2 caused DA neurons to gradually lose their dopaminergic phenotype. Similar down-regulation of Aldh1a1, TH, DAT, and AADC was also reported in post-mitotic DA neurons after prenatal ablation of Foxa1/2 (Stott et al., 2013) that is in a good agreement with our data. However, in that study, the authors also detected a decrease in TH-positive neurons both pre- and postnatally. On the contrary, in our model, when inactivation of both factors occurred in adult DA neurons, we did not detect any loss of TH-positive cells up to 24 weeks post-TAM. These data suggest that Foxa1/2 factors are essential for DA survival during their maturation and specification, while, similarly to PD, additional epigenetic cues (such as environmental factors, mitochondrial stress or aging) are required for the onset of neurodegeneration when Foxa factors are inactivated in mature DA neurons.

Foxa2 can regulate TH expression directly and/or cooperatively with Nurr1 (Lee et al., 2010), and there are three Foxa2 binding sites in the promoter region of the mouse *Aadc* gene identified by ChIP (Soccio et al., 2011; Yang et al., 2013). It has also been shown that Foxa1/2 loss leads to lower occupancy of *Aadc* and *Th* gene promoters by Nurr1 that, without affecting the levels of Nurr1 itself, results in the down-regulation of AADC in post-mitotic DA neurons (Stott et al., 2013). Of note, the loss of Foxa1/2 did not lead to down-regulation of the levels of either Nurr1 or several other factors important for the functions of DA neurons in the ventral midbrain (Supplementary Figure 2). However, consistent with the role of Foxa proteins as “pioneer” factors that increase chromatin accessibility for other transcriptional regulators (Friedman and Kaestner, 2006), the loss of Foxa1/2 might have affected the ability of other transcription factors, including Nurr1, to bind their target promoters in adult DA neurons, as it was observed in a study with the prenatal ablation of Foxa1/2 factors (Stott et al., 2013).

By mining the available gene expression profiling data, we have found that the expression levels of *Foxa1* and *Foxa2*, as well as *Aldh1a1, Nr4a2, Aadc, Th, En1*, and *Slc6a3* were also down-regulated in the SNpc, but not in other brain regions or tissue samples from PD patients. However, the profiling data from patients' SNpc samples should be interpreted cautiously, because the apparent down-regulation of these genes might just reflect the loss of DA neurons expressing them. Nevertheless, in the context of our results and previously published data (Kittappa et al., 2007; Stott et al., 2013), the observed down-regulation of Foxa1/2 in post-mortem samples from PD patients suggests that Foxa1/2 factors and their target genes may have a specific role in the SNpc and contribute to neurodegeneration in PD patients.

In summary, we show that, similar to their role in the embryonic development (Ferri et al., 2007; Stott et al., 2013), Foxa1 can compensate for the loss of Foxa2 in adult DA neurons. Thus, a functional redundancy between Foxa1 and Foxa2 proteins, initially observed during embryonic development, is also evident in adult DA neurons. We have further demonstrated that the ablation of Foxa factors in adult DA neurons initially led to the loss of Aldh1a1 expression accompanied by the loss of striatal dopamine and locomotor impairments in the rotarod and open field tests. Foxa factors may regulate *Aldh1a1* directly by binding to the gene's promoter (Soccio et al., 2011; Yang et al., 2013) and/or indirectly by opening chromatin and facilitating the binding of other transcription factors (Friedman and Kaestner, 2006). This data suggests that Foxa1/2 ablation led to the loss of dopaminergic phenotype in SNpc DA neurons that was further confirmed by the observed down-regulation of AADC and DAT expression in the SNpc. Ultimately, we detected a significant loss of TH-positive DA neurons in aged *cFoxa1/2*^−/−^ mice (Figures 5B,C), resembling the course of events during the PD pathology in humans.

Altogether, our data establish a protective role of Foxa factors in the maintenance of dopamine neurons *in vivo*. Drugs targeting cytoprotective pathways in DA neurons of human patients with PD are already effectively used or being tested in clinical studies (Allain et al., 2008; Youdim, 2010; Pahwa and Lyons, 2014). The transcription factors from the Foxa family could become additional candidates for such therapeutic strategies.

### Conflict of interest statement

The authors declare that the research was conducted in the absence of any commercial or financial relationships that could be construed as a potential conflict of interest.

## Abbreviations

AADC: aromatic amino acid decarboxylase
Aldh1a1: aldehyde dehydrogenase family 1, subfamily A1
ChIP: chromatin immunoprecipitation
DA: dopamine
DAT: dopamine transporter
DOPAC: 3,4-dihydroxyphenylacetic acid
DOPAL: 3,4-dihydroxyphenylacetaldehyde
En1: engrailed 1
HPLC-ED: high-performance liquid chromatography with electrochemical detection
HVA: homovanillic acid
MPTP: 1-methyl-4-phenyl-1,2,3,6-tetrahydropyridine
PD: Parkinson's disease
post-TAM: after tamoxifen treatment
rpm: rounds per minute
s.e.m.: standard error of means
SNpc: substantia nigra pars compacta
TAM: tamoxifen
TH: tyrosine hydroxylase
VTA: ventral tegmental area.
