# Supplementary material for: Transcription factors Foxa1 and Foxa2 are required for adult dopamine neurons maintenance
Source: Front Cell Neurosci. 2014 Sep 9;8:275. doi: 10.3389/fncel.2014.00275 (PMC4158790; doi:10.3389/fncel.2014.00275)
Supplement: Supplementary file 1 [file DataSheet1.DOCX]

***Supplementary Material***

**Transcription factors Foxa1 and Foxa2 are required for adult dopamine neurons maintenance**

**Andrii Domanskyi^1#^, Heike Alter^1^, Miriam A. Vogt^2^, Peter Gass^2^, Ilya A. Vinnikov^1^***

^1^Division of Molecular Biology of the Cell I, German Cancer Research Center (DKFZ), Heidelberg, Germany

^2^RG Animal Models in Psychiatry, Central Institute of Mental Health, Medical Faculty Mannheim, Heidelberg University, Mannheim, Germany

*** Correspondence:** Ilya A. Vinnikov, Division of Molecular Biology of the Cell I, German Cancer Research Center (DKFZ), Im Neuenheimer Feld 280, 69120 Heidelberg, Germany

[ilya.vinnikov@gmail.com](mailto:ilya.vinnikov@gmail.com)

# Current address: Institute of Biotechnology, University of Helsinki, Helsinki, Finland

**Supplementary Figures**


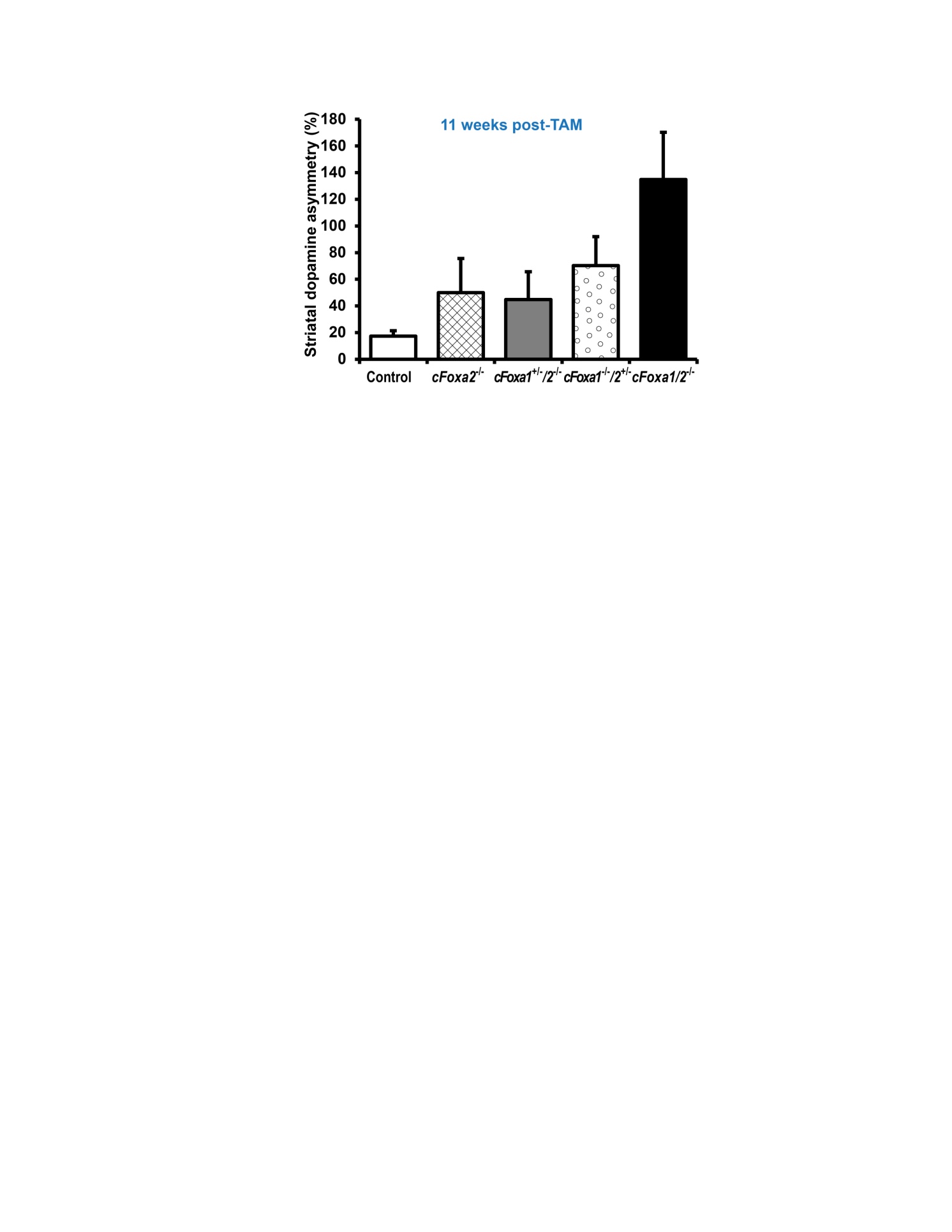


**Supplementary Figure 1. Left-right asymmetry in the striatal dopamine content in *cFoxa1/2* mice.** Relative difference in the striatal dopamine content in left and right hemispheres in mice 11 weeks after conditional ablation of Foxa1 and/or Foxa2 in adult dopamine neurons.


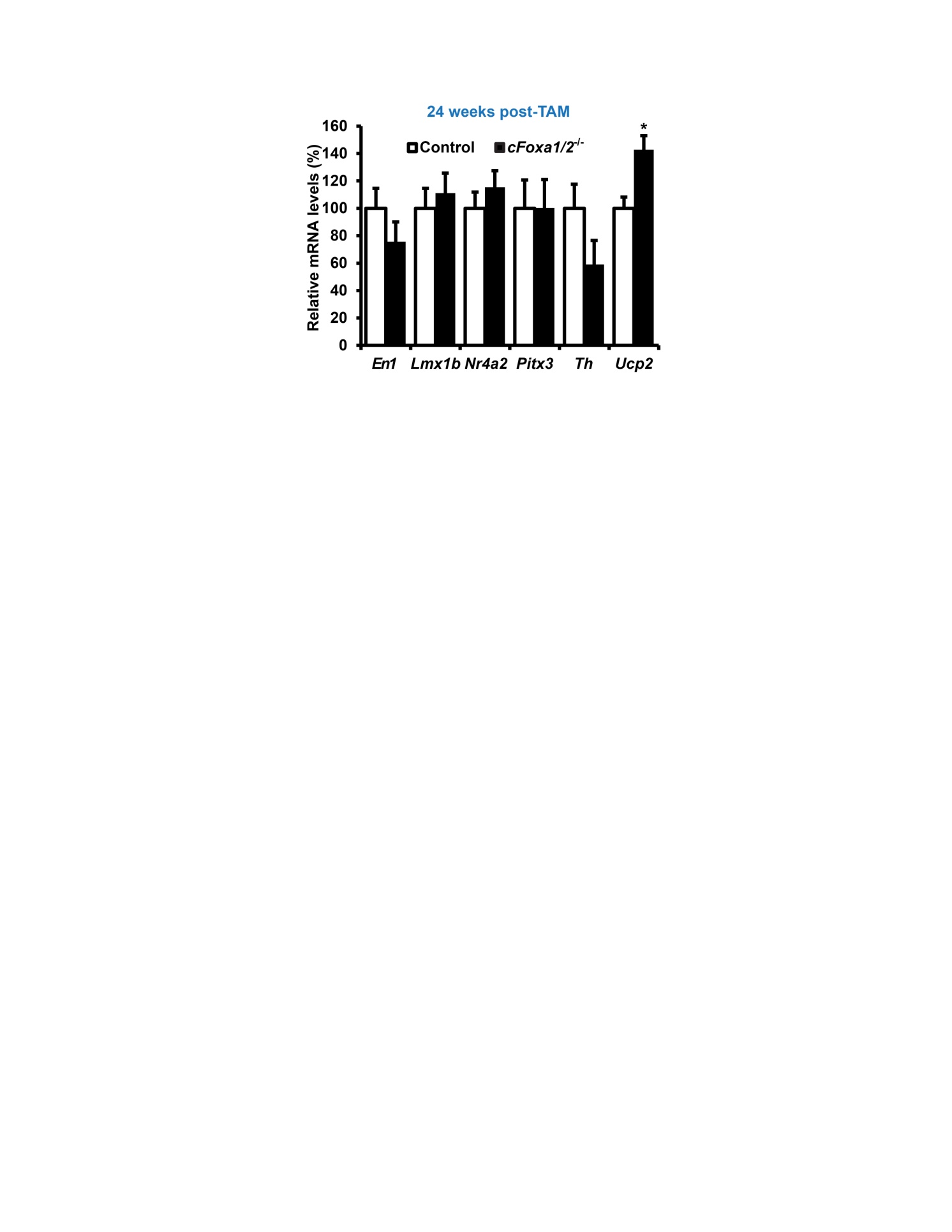


**Supplementary Figure 2. Levels of transcripts in the ventral midbrain of *cFoxa1/2^-/-^* mice.** Relative mRNA levels of factors involved in the development and functionality of dopamine neurons in the ventral midbrain of *cFoxa2*^-/-^ mice measured by quantitative PCR 24 weeks after tamoxifen treatment (post-TAM). *, *p<*0.05, in comparison to control, as determined by Student’s unpaired *t*-test.
